# 2,6-Diamino-4-chloro­pyrimidine–benzoic acid (1/1)

**DOI:** 10.1107/S160053681204768X

**Published:** 2012-11-24

**Authors:** Kaliyaperumal Thanigaimani, Nuridayanti Che Khalib, Suhana Arshad, Ibrahim Abdul Razak

**Affiliations:** a School of Physics, Universiti Sains Malaysia, 11800 USM, Penang, Malaysia

## Abstract

The benzoic acid mol­ecule of the title compound, C_4_H_5_ClN_4_·C_7_H_6_O_2_, is approximately planar, with a dihedral angle of 1.28 (9)° between the carb­oxy group and the benzene ring. In the crystal, two acid and two base mol­ecules are linked through N—H⋯O and O—H⋯N hydrogen bonds, forming a centrosymmetric 2 + 2 unit with *R*_2_^2^(8) and *R*_4_^2^(8) motifs. These units are further linked through a pair of N—H⋯N hydrogen bonds into a tape structure along [1-20]. The crystal structure also features weak π–π [centroid–centroid distance = 3.5984 (11) Å] and C—H⋯π inter­actions.

## Related literature

For the biological activity of pyrimidine and amino­pyrimidine derivatives, see: Hunt *et al.* (1980 ▶); Baker & Santi (1965 ▶). For related structures, see: Schwalbe & Williams (1982 ▶); Hu *et al.* (2002 ▶); Chinnakali *et al.* (1999 ▶); Skovsgaard & Bond (2009 ▶). For hydrogen-bond motifs, see: Bernstein *et al.* (1995 ▶). For bond-length data, see: Allen *et al.* (1987 ▶). For the stability of the temperature controller used for the data collection, see: Cosier & Glazer (1986 ▶).
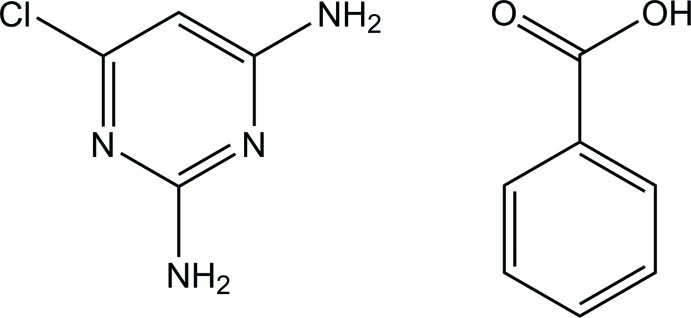


## Experimental

### 

#### Crystal data

C_4_H_5_ClN_4_·C_7_H_6_O_2_
*M* *_r_* = 266.69
Monoclinic, 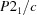

*a* = 8.7817 (17) Å
*b* = 5.7032 (12) Å
*c* = 24.026 (4) Å
β = 95.493 (4)°
*V* = 1197.8 (4) Å^3^
*Z* = 4
Mo *K*α radiation
μ = 0.32 mm^−1^
*T* = 100 K
0.36 × 0.30 × 0.16 mm

#### Data collection

Bruker SMART APEXII CCD area-detector diffractometer
Absorption correction: multi-scan (*SADABS*; Bruker, 2009 ▶) *T*_min_ = 0.895, *T*_max_ = 0.951
7539 measured reflections
2097 independent reflections
1891 reflections with *I* > 2σ(*I*)
*R*_int_ = 0.052

#### Refinement

*R*[*F* ^2^ > 2σ(*F* ^2^)] = 0.036
*wR*(*F* ^2^) = 0.101
*S* = 1.09
2097 reflections
183 parameters
1 restraint
H atoms treated by a mixture of independent and constrained refinement
Δρ_max_ = 0.25 e Å^−3^
Δρ_min_ = −0.24 e Å^−3^

### 

Data collection: *APEX2* (Bruker, 2009 ▶); cell refinement: *SAINT* (Bruker, 2009 ▶); data reduction: *SAINT*; program(s) used to solve structure: *SHELXTL* (Sheldrick, 2008 ▶); program(s) used to refine structure: *SHELXTL*; molecular graphics: *SHELXTL*; software used to prepare material for publication: *SHELXTL* and *PLATON* (Spek, 2009 ▶).

## Supplementary Material

Crystal structure: contains datablock(s) global, I. DOI: 10.1107/S160053681204768X/is5218sup1.cif

Structure factors: contains datablock(s) I. DOI: 10.1107/S160053681204768X/is5218Isup2.hkl

Supplementary material file. DOI: 10.1107/S160053681204768X/is5218Isup3.cml

Additional supplementary materials:  crystallographic information; 3D view; checkCIF report

## Figures and Tables

**Table 1:** Hydrogen-bond geometry (Å, °) *Cg*1 is the centroid of the C5–C10 ring.

*D*—H⋯*A*	*D*—H	H⋯*A*	*D*⋯*A*	*D*—H⋯*A*
O1—H1*O*1⋯N2	0.87 (2)	1.74 (2)	2.5976 (18)	168 (3)
N4—H2*N*4⋯O2	0.88 (2)	2.03 (2)	2.894 (2)	171.2 (18)
N4—H1*N*4⋯O2^i^	0.88 (2)	2.07 (2)	2.902 (2)	158.2 (19)
N3—H1*N*3⋯N1^ii^	0.85 (2)	2.18 (2)	3.020 (2)	171 (2)
C9—H9*A*⋯*Cg*1^iii^	0.95	2.99	3.6557 (19)	128

Symmetry codes: (i) 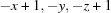; (ii) 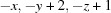; (iii) 
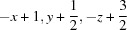
.

**Fig. 1.**
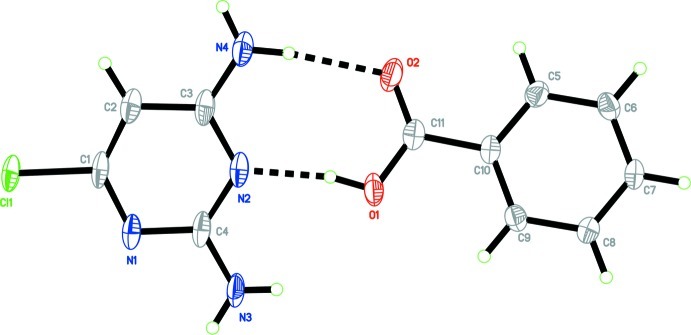
The molecular structure of the title compound with atom labels with 50% probability displacement ellipsoids. Dashed lines indicate the hydrogen bonds.

**Fig. 2.**
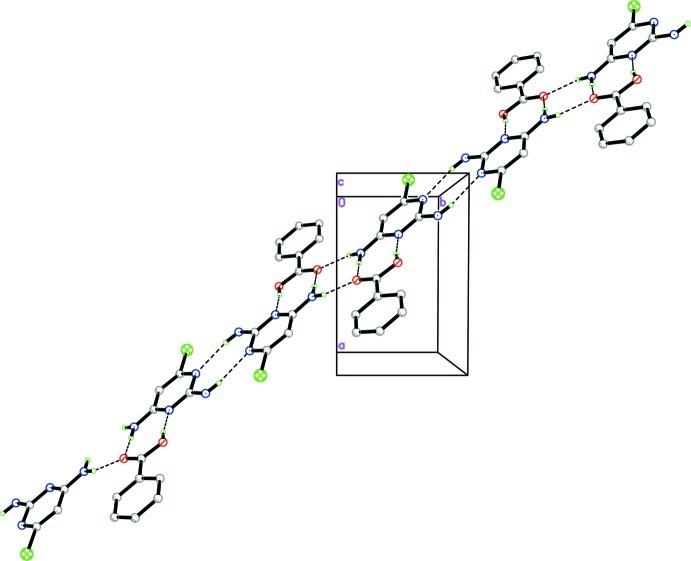
The crystal packing of the title compound. H atoms not involved in the intermolecular interactions (dashed lines) have been omitted for clarity.

## supplementary crystallographic information

### Comment

Pyrimidine and aminopyrimidine derivatives are biologically important compounds as they occur in nature as components of nucleic acids. Some aminopyrimidine derivatives are used as antifolate drugs (Hunt *et al.*, 1980; Baker & Santi, 1965). The crystal structures of aminopyrimidine derivatives (Schwalbe & Williams, 1982), aminopyrimidine carboxylates (Hu *et al.*, 2002) and co-crystal structures (Chinnakali *et al.*, 1999; Skovsgaard & Bond, 2009) have ben reported. In the present study, hydrogen-bonding patterns in the 2,6-diamino-4-chloropyrimidine–benzoic acid (1/1) co-crystal are investigated.

The asymmetric unit (Fig. 1) contains one 2,6-diamino-4-chloropyrimidine molecule and one benzoic acid molecule. The 2,6-diamino-4-chloropyrimidine molecule is essentially planar, with a maximum deviation of 0.009 (2) Å for atom C4. The carboxyl group of the benzoic acid molecule is twisted slightly from the ring with a dihedral angle between C5–C10 ring and O1/O2/C10/C11 plane being 1.28 (9)°. The bond lengths (Allen *et al.*, 1987) and angles are normal.

In the crystal packing (Fig. 2), the 2,6-diamino-4-chloropyrimidine molecules interact with the carboxylic group of the respective benzoic acid molecules through N4—H2N4···O2 and O1—H1O1···N2 hydrogen bonds, forming a cyclic hydrogen-bonded motif of *R*_2_^2^(8) (Bernstein *et al.*, 1995). These motifs are centrosymmetrically paired *via* N4—H1N4···O2^i^ hydrogen bonds, resulting in a DADA array (Where D is a hydrogen-bond donor and A is a hydrogen-bond acceptor) of quadruple hydrogen bonds (symmetry code in Table 1); this can be represented by the graph-set notations of *R*_2_^2^(8) and *R*_4_^2^(8). The quadruple hydrogen-bonding motifs are further extended through a couple of N3—H1N3···N1^ii^ hydrogen bonds (symmetry code in Table 1), leading to the formation of hydrogen-bonded supramolecular tape. The crystal structure is further stabilized by π–π interactions between the pyrimidine (*Cg*2; N1/N2/C1–C4) rings [*Cg*2···*Cg*2 = 3.5984 (11) Å; -*x*, 1 - *y*, 1 - *z*] and C—H···π interactions (Table 1) involving the centroid of the C5–C10 (centroid *Cg*1) ring.

### Experimental

A hot methanol solutions (20 ml) of 2,6-diamino-4-chloropyrimidine (36 mg, Aldrich) and benzoic acid (30 mg, Merck) were mixed and warmed over a heating magnetic stirrer hotplate for a few minutes. The resulting solution was allowed to cool slowly at room temperature and crystals of the title compound (I) appeared after a few days.

### Refinement

O- and N-bound H Atoms were located in a difference Fourier maps and refined freely [O—H = 0.866 (10) Å and N—H = 0.79 (2)–0.88 (2) Å]. The remaining hydrogen atoms were positioned geometrically (C—H = 0.95 Å) and were refined using a riding model, with *U*_iso_(H) = 1.2 *U*_eq_(C).

### Crystal data

**Table:**

C_4_H_5_ClN_4_·C_7_H_6_O_2_	*F*(000) = 552
*M**_r_* = 266.69	*D*_x_ = 1.479 Mg m^−^^3^
Monoclinic, *P*2_1_/*c*	Mo *K*α radiation, λ = 0.71073 Å
Hall symbol: -P 2ybc	Cell parameters from 5646 reflections
*a* = 8.7817 (17) Å	θ = 3.0–30.0°
*b* = 5.7032 (12) Å	µ = 0.32 mm^−^^1^
*c* = 24.026 (4) Å	*T* = 100 K
β = 95.493 (4)°	Block, colourless
*V* = 1197.8 (4) Å^3^	0.36 × 0.30 × 0.16 mm
*Z* = 4	

### Data collection

**Table:**

Bruker SMART APEXII CCD area-detector diffractometer	2097 independent reflections
Radiation source: fine-focus sealed tube	1891 reflections with *I* > 2σ(*I*)
Graphite monochromator	*R*_int_ = 0.052
φ and ω scans	θ_max_ = 25.0°, θ_min_ = 2.3°
Absorption correction: multi-scan (*SADABS*; Bruker, 2009)	*h* = −10→10
*T*_min_ = 0.895, *T*_max_ = 0.951	*k* = −6→6
7539 measured reflections	*l* = −28→28

### Refinement

**Table:**

Refinement on *F*^2^	Primary atom site location: structure-invariant direct methods
Least-squares matrix: full	Secondary atom site location: difference Fourier map
*R*[*F*^2^ > 2σ(*F*^2^)] = 0.036	Hydrogen site location: inferred from neighbouring sites
*wR*(*F*^2^) = 0.101	H atoms treated by a mixture of independent and constrained refinement
*S* = 1.09	*w* = 1/[σ^2^(*F*_o_^2^) + (0.0471*P*)^2^ + 0.4196*P*] where *P* = (*F*_o_^2^ + 2*F*_c_^2^)/3
2097 reflections	(Δ/σ)_max_ < 0.001
183 parameters	Δρ_max_ = 0.25 e Å^−^^3^
1 restraint	Δρ_min_ = −0.24 e Å^−^^3^

### Special details

**Table:**

Experimental. The crystal was placed in the cold stream of an Oxford Cryosystems Cobra open-flow nitrogen cryostat (Cosier & Glazer, 1986) operating at 100.0 (1) K.
Geometry. All e.s.d.'s (except the e.s.d. in the dihedral angle between two l.s. planes) are estimated using the full covariance matrix. The cell e.s.d.'s are taken into account individually in the estimation of e.s.d.'s in distances, angles and torsion angles; correlations between e.s.d.'s in cell parameters are only used when they are defined by crystal symmetry. An approximate (isotropic) treatment of cell e.s.d.'s is used for estimating e.s.d.'s involving l.s. planes.
Refinement. Refinement of *F*^2^ against ALL reflections. The weighted *R*-factor *wR* and goodness of fit *S* are based on *F*^2^, conventional *R*-factors *R* are based on *F*, with *F* set to zero for negative *F*^2^. The threshold expression of *F*^2^ > σ(*F*^2^) is used only for calculating *R*-factors(gt) *etc*. and is not relevant to the choice of reflections for refinement. *R*-factors based on *F*^2^ are statistically about twice as large as those based on *F*, and *R*- factors based on ALL data will be even larger.

### Fractional atomic coordinates and isotropic or equivalent isotropic displacement parameters (Å^2^)

**Table:**

	*x*	*y*	*z*	*U*_iso_*/*U*_eq_	
Cl1	−0.05557 (4)	0.64565 (9)	0.367228 (16)	0.03348 (18)	
O1	0.43197 (13)	0.5175 (2)	0.61239 (5)	0.0312 (3)	
O2	0.52962 (16)	0.1720 (2)	0.59015 (5)	0.0372 (3)	
N1	0.07033 (15)	0.7543 (3)	0.46563 (5)	0.0274 (4)	
N2	0.27154 (14)	0.5362 (3)	0.51588 (5)	0.0266 (4)	
N3	0.16941 (19)	0.8669 (3)	0.55310 (6)	0.0313 (4)	
N4	0.37676 (16)	0.2112 (3)	0.47835 (7)	0.0325 (4)	
C1	0.07703 (17)	0.5991 (3)	0.42471 (7)	0.0272 (4)	
C2	0.17389 (17)	0.4136 (4)	0.42399 (7)	0.0287 (4)	
H2A	0.1734	0.3108	0.3929	0.034*	
C3	0.27541 (17)	0.3846 (3)	0.47284 (7)	0.0272 (4)	
C4	0.17144 (17)	0.7163 (3)	0.51057 (6)	0.0265 (4)	
C5	0.71603 (19)	0.1587 (3)	0.69200 (7)	0.0264 (4)	
H5A	0.7220	0.0302	0.6672	0.032*	
C6	0.80722 (19)	0.1626 (3)	0.74236 (7)	0.0276 (4)	
H6A	0.8760	0.0372	0.7519	0.033*	
C7	0.79790 (17)	0.3497 (3)	0.77877 (7)	0.0251 (4)	
H7A	0.8608	0.3526	0.8132	0.030*	
C8	0.69723 (17)	0.5321 (3)	0.76507 (7)	0.0258 (4)	
H8A	0.6908	0.6594	0.7902	0.031*	
C9	0.60544 (17)	0.5293 (3)	0.71455 (7)	0.0241 (4)	
H9A	0.5361	0.6542	0.7052	0.029*	
C10	0.61545 (17)	0.3430 (3)	0.67768 (6)	0.0214 (4)	
C11	0.52134 (18)	0.3366 (3)	0.62273 (7)	0.0249 (4)	
H2N3	0.233 (3)	0.851 (4)	0.5785 (10)	0.039 (6)*	
H2N4	0.432 (2)	0.196 (4)	0.5105 (10)	0.045 (6)*	
H1N4	0.388 (2)	0.113 (4)	0.4508 (9)	0.033 (5)*	
H1N3	0.109 (2)	0.983 (4)	0.5499 (9)	0.037 (6)*	
H1O1	0.386 (3)	0.508 (7)	0.5789 (7)	0.113 (13)*	

### Atomic displacement parameters (Å^2^)

**Table:**

	*U*^11^	*U*^22^	*U*^33^	*U*^12^	*U*^13^	*U*^23^
Cl1	0.0286 (3)	0.0508 (4)	0.0183 (2)	−0.00328 (18)	−0.01233 (16)	0.00310 (18)
O1	0.0260 (6)	0.0437 (8)	0.0220 (6)	0.0042 (6)	−0.0080 (5)	0.0035 (6)
O2	0.0521 (8)	0.0321 (8)	0.0240 (6)	−0.0019 (6)	−0.0147 (6)	−0.0010 (6)
N1	0.0230 (6)	0.0382 (9)	0.0190 (7)	−0.0093 (6)	−0.0076 (5)	0.0077 (7)
N2	0.0203 (6)	0.0395 (9)	0.0183 (7)	−0.0085 (6)	−0.0059 (5)	0.0068 (6)
N3	0.0318 (8)	0.0370 (10)	0.0217 (8)	−0.0044 (7)	−0.0144 (6)	0.0040 (7)
N4	0.0262 (7)	0.0503 (11)	0.0195 (7)	−0.0020 (7)	−0.0058 (6)	0.0009 (7)
C1	0.0202 (7)	0.0439 (11)	0.0159 (8)	−0.0113 (7)	−0.0071 (6)	0.0080 (7)
C2	0.0218 (8)	0.0457 (12)	0.0177 (8)	−0.0074 (8)	−0.0032 (6)	0.0029 (8)
C3	0.0178 (7)	0.0447 (12)	0.0181 (8)	−0.0098 (7)	−0.0029 (6)	0.0071 (7)
C4	0.0214 (7)	0.0387 (11)	0.0178 (8)	−0.0124 (7)	−0.0062 (6)	0.0083 (7)
C5	0.0325 (9)	0.0257 (10)	0.0201 (8)	0.0009 (7)	−0.0015 (7)	−0.0003 (7)
C6	0.0283 (8)	0.0292 (10)	0.0244 (8)	0.0070 (7)	−0.0030 (7)	0.0050 (7)
C7	0.0220 (8)	0.0322 (10)	0.0196 (8)	−0.0019 (7)	−0.0053 (6)	0.0024 (7)
C8	0.0249 (8)	0.0268 (10)	0.0244 (8)	−0.0006 (7)	−0.0038 (6)	−0.0034 (7)
C9	0.0199 (7)	0.0248 (10)	0.0267 (8)	0.0005 (7)	−0.0027 (6)	0.0028 (7)
C10	0.0192 (7)	0.0263 (9)	0.0181 (8)	−0.0046 (6)	−0.0014 (6)	0.0039 (6)
C11	0.0243 (8)	0.0291 (10)	0.0205 (8)	−0.0063 (7)	−0.0023 (6)	0.0043 (7)

### Geometric parameters (Å, º)

**Table:**

Cl1—C1	1.7395 (15)	C2—C3	1.414 (2)
O1—C11	1.306 (2)	C2—H2A	0.9500
O1—H1O1	0.866 (10)	C5—C6	1.386 (2)
O2—C11	1.229 (2)	C5—C10	1.395 (2)
N1—C1	1.328 (2)	C5—H5A	0.9500
N1—C4	1.348 (2)	C6—C7	1.387 (3)
N2—C4	1.350 (2)	C6—H6A	0.9500
N2—C3	1.351 (2)	C7—C8	1.384 (2)
N3—C4	1.336 (2)	C7—H7A	0.9500
N3—H2N3	0.79 (2)	C8—C9	1.392 (2)
N3—H1N3	0.85 (2)	C8—H8A	0.9500
N4—C3	1.328 (3)	C9—C10	1.391 (2)
N4—H2N4	0.88 (2)	C9—H9A	0.9500
N4—H1N4	0.88 (2)	C10—C11	1.490 (2)
C1—C2	1.358 (3)		
			
C11—O1—H1O1	110 (2)	C6—C5—C10	120.18 (16)
C1—N1—C4	114.40 (16)	C6—C5—H5A	119.9
C4—N2—C3	118.60 (14)	C10—C5—H5A	119.9
C4—N3—H2N3	117.1 (16)	C5—C6—C7	119.92 (16)
C4—N3—H1N3	119.2 (15)	C5—C6—H6A	120.0
H2N3—N3—H1N3	123 (2)	C7—C6—H6A	120.0
C3—N4—H2N4	118.1 (15)	C8—C7—C6	120.20 (15)
C3—N4—H1N4	121.4 (13)	C8—C7—H7A	119.9
H2N4—N4—H1N4	121 (2)	C6—C7—H7A	119.9
N1—C1—C2	126.90 (15)	C7—C8—C9	120.15 (16)
N1—C1—Cl1	114.35 (13)	C7—C8—H8A	119.9
C2—C1—Cl1	118.76 (14)	C9—C8—H8A	119.9
C1—C2—C3	115.25 (17)	C10—C9—C8	119.83 (15)
C1—C2—H2A	122.4	C10—C9—H9A	120.1
C3—C2—H2A	122.4	C8—C9—H9A	120.1
N4—C3—N2	117.76 (15)	C9—C10—C5	119.71 (15)
N4—C3—C2	122.23 (17)	C9—C10—C11	121.29 (15)
N2—C3—C2	120.01 (17)	C5—C10—C11	118.99 (15)
N3—C4—N1	116.94 (17)	O2—C11—O1	123.62 (15)
N3—C4—N2	118.24 (15)	O2—C11—C10	121.44 (16)
N1—C4—N2	124.81 (16)	O1—C11—C10	114.94 (15)
			
C4—N1—C1—C2	−0.5 (2)	C10—C5—C6—C7	0.3 (3)
C4—N1—C1—Cl1	179.02 (11)	C5—C6—C7—C8	0.3 (3)
N1—C1—C2—C3	1.1 (3)	C6—C7—C8—C9	−0.4 (2)
Cl1—C1—C2—C3	−178.45 (11)	C7—C8—C9—C10	−0.2 (2)
C4—N2—C3—N4	178.65 (15)	C8—C9—C10—C5	0.9 (2)
C4—N2—C3—C2	−1.2 (2)	C8—C9—C10—C11	−178.71 (14)
C1—C2—C3—N4	179.98 (15)	C6—C5—C10—C9	−0.9 (2)
C1—C2—C3—N2	−0.1 (2)	C6—C5—C10—C11	178.67 (15)
C1—N1—C4—N3	−179.82 (14)	C9—C10—C11—O2	−179.79 (15)
C1—N1—C4—N2	−1.0 (2)	C5—C10—C11—O2	0.6 (2)
C3—N2—C4—N3	−179.31 (15)	C9—C10—C11—O1	0.5 (2)
C3—N2—C4—N1	1.9 (2)	C5—C10—C11—O1	−179.08 (14)

### Hydrogen-bond geometry (Å, º)

*Cg*1 is the centroid of the C5–C10 ring.

**Table:**

*D*—H···*A*	*D*—H	H···*A*	*D*···*A*	*D*—H···*A*
O1—H1*O*1···N2	0.87 (2)	1.74 (2)	2.5976 (18)	168 (3)
N4—H2*N*4···O2	0.88 (2)	2.03 (2)	2.894 (2)	171.2 (18)
N4—H1*N*4···O2^i^	0.88 (2)	2.07 (2)	2.902 (2)	158.2 (19)
N3—H1*N*3···N1^ii^	0.85 (2)	2.18 (2)	3.020 (2)	171 (2)
C9—H9*A*···*Cg*1^iii^	0.95	2.99	3.6557 (19)	128

Symmetry codes: (i) −*x*+1, −*y*, −*z*+1; (ii) −*x*, −*y*+2, −*z*+1; (iii) −*x*+1, *y*+1/2, −*z*+3/2.

## References

[bb1] Allen, F. H., Kennard, O., Watson, D. G., Brammer, L., Orpen, A. G. & Taylor, R. (1987). *J. Chem. Soc. Perkin Trans. 2*, pp. S1–19.

[bb2] Baker, B. R. & Santi, D. V. (1965). *J. Pharm. Sci.* **54**, 1252–1257. 10.1002/jps.2600540905 5881214

[bb3] Bernstein, J., Davis, R. E., Shimoni, L. & Chang, N.-L. (1995). *Angew. Chem. Int. Ed. Engl.* **34**, 1555–1573.

[bb4] Bruker (2009). *SADABS*, *APEX2* and *SAINT* Bruker AXS Inc., Madison, Wisconsin, USA.

[bb5] Chinnakali, K., Fun, H.-K., Goswami, S., Mahapatra, A. K. & Nigam, G. D. (1999). *Acta Cryst.* C**55**, 399–401.

[bb6] Cosier, J. & Glazer, A. M. (1986). *J. Appl. Cryst.* **19**, 105–107.

[bb7] Hu, M.-L., Ye, M.-D., Zain, S. M. & Ng, S. W. (2002). *Acta Cryst.* E**58**, o1005–o1007.

[bb8] Hunt, W. E., Schwalbe, C. H., Bird, K. & Mallinson, P. D. (1980). *J. Biochem.* **187**, 533–536. 10.1042/bj1870533 PMC1161822 6893149

[bb9] Schwalbe, C. H. & Williams, G. J. B. (1982). *Acta Cryst.* B**38**, 1840–1843.

[bb10] Sheldrick, G. M. (2008). *Acta Cryst.* A**64**, 112–122. 10.1107/S0108767307043930 18156677

[bb11] Skovsgaard, S. & Bond, A. D. (2009). *CrystEngComm*, **11**, 444–453.

[bb12] Spek, A. L. (2009). *Acta Cryst.* D**65**, 148–155. 10.1107/S090744490804362X PMC2631630 19171970

